# Prevalence and Risk Factors for Unhealthy Dietary Habits Among School Children in Hong Kong During COVID‐19 Pandemic: A Cross‐Sectional Study

**DOI:** 10.1002/hsr2.71746

**Published:** 2026-01-19

**Authors:** Junjie Huang, Sze Chai Chan, Wing Sze Pang, Fung Yu Mak, Yat Ching Fung, Vera M. W. Keung, Calvin K. M. Cheung, Vincent T. C. Lau, Amelia S. C. Lo, Claire Chenwen Zhong, Lancelot W. H. Mui, Albert Lee, Martin C. S. Wong

**Affiliations:** ^1^ Centre for Health Education and Health Promotion, Faculty of Medicine The Chinese University of Hong Kong Shatin Hong Kong SAR China; ^2^ Jockey Club School of Public Health and Primary Care, Faculty of Medicine The Chinese University of Hong Kong Shatin Hong Kong SAR China; ^3^ The School of Public Health, Peking University Beijing China; ^4^ The School of Public Health, The Chinese Academy of Medical Sciences and The Peking Union Medical Colleges Beijing China; ^5^ The School of Public Health, Fudan University Shanghai China

**Keywords:** COVID‐19 pandemic, obesity, risk factor, unhealthy dietary habits

## Abstract

**Background and Aims:**

Evidence demonstrates unhealthy dietary patterns in early life may contribute to obesity and increased risk of chronic diseases in later life. The COVID‐19 pandemic has the potential to impact the dietary habits of schoolchildren due to movement restrictions. Therefore, this study aims to investigate the prevalence of unhealthy dietary habits among primary and secondary school students in Hong Kong during COVID‐19, along with associated factors.

**Methods:**

A cross‐sectional study was conducted among the primary and secondary school students in Hong Kong from September 2021 to November 2021. Data on sociodemographic information, dietary habits, and lifestyle were collected using self‐administered questionnaires. Multivariate logistic regression was conducted to investigate the association between variables and unhealthy dietary habits.

**Results:**

A total of 1541 participants were included, with 762 primary school students (mean age: 10.0) and 779 secondary school students (mean age: 13.6). Approximately 81.5% of primary school students and 89.5% of secondary school students reported inadequate intake of vegetables and fruits. 18.6% of primary students and 42.8% of secondary students reported skipping breakfast, while 46.4% of primary students and 49.2% of secondary students consumed unhealthy foods. Analysis indicates that inadequate vegetable and fruit intake was positively associated with physical inactivity in both groups (aORs = 3.26–3.39). Students who engage in excessive screen time on games or social media had higher odds of skipping breakfast and consuming unhealthy foods (aOR = 1.47–2.24). Secondary school students who perceived themselves as underweight had higher odds of consuming unhealthy foods (aOR = 1.81), while those who reported being overweight had higher odds of skipping breakfast (aOR = 1.51).

**Conclusion:**

Findings highlighted the high prevalence of unhealthy dietary habits among school children in Hong Kong and identified physical inactivity and excessive screen time as key contributing factors. Future research should develop and validate interventions to improve dietary habits.

## 1 Introduction

In March 2020, Covid‐19 was declared as a global pandemic by the World Health Organization (WHO) [1]. At the beginning of the outbreak, no vaccine was available to prevent infections. As a result, various policies were implemented to curtail the spread of the virus. To limit interpersonal contact, governments imposed restrictions on public access to different facilities and implemented lockdown measures that included supermarkets and schools [2]. The lockdowns had a significant impact on food supply, particularly fresh food, and consequently, food purchases and eating habits were affected.

A healthy diet is crucial for the growth, development, and general health of children. Unhealthy dietary habits, such as consuming high‐calorie food, sugary drinks, and processed food, can increase the risk of being overweight, obese, and developing chronic diseases [3]. However, the COVID‐19 pandemic has posed significant challenges to the dietary behaviors of schoolchildren. The socio‐ecological model emphasizes that behavior development involves not only individual‐level factors but also the interconnectedness of interpersonal, organizational, community, and policy levels [4]. The influence introduced by the change in daily life, such as school closures, increased time spent indoors, and disruptions to daily routines, during the COVID‐19 pandemic, warrants attention regarding changes in children's eating habits [5]. Likewise, children may be more prone to adopting unhealthy lifestyle habits due to limited access to healthy food choices, decreased physical exercise, and increased screen time [6].

The COVID‐19 pandemic has had a significant impact on the dietary and lifestyle choices of children and adolescents. The closure of schools and the implementation of social distancing measures have forced many children to stay at home for extended periods, with limited access to physical activity and healthy food options. However, in Hong Kong, no studies have yet been conducted to explore the dietary habits of schoolchildren during the COVID‐19 pandemic, to inform post‐pandemic policy‐making. Therefore, this study aims to find out the prevalence of unhealthy eating habits, including inadequate fruit and vegetable intake, unhealthy food consumption, and skipping breakfast, among primary and secondary school students, as well as factors associated with them.

## 2 Methodology

### 2.1 Ethical Approval

This study was approved by the Survey and Behavioral Research Ethics Committee, The Chinese University of Hong Kong (No. SBRE‐21‐0052). Informed consents were obtained from all participants.

### 2.2 Study Design

This is a cross‐sectional study among primary and secondary school students in Hong Kong, conducted between September 2021 and November 2021. A cluster sampling approach was employed to recruit schools that participated in the GoSmart.Net Built‐on Project, resulting in a total of 21 primary and secondary schools (20 were public and 1 was private) being included and covering 12 out of 18 districts in Hong Kong, providing a similar distribution with Hong Kong's overall school system (approximately 90% public and 10% private). We estimated that a minimum of 601 participants is required for the primary school student cohort and the secondary school student cohort, respectively, under the assumption that 50% of participants report outcomes of interest (proportion p), with 5% type I error rate and achieve a precision level of 0.04 based on the formular: [N = 1.96 × p(1–p)/precision^2^] [7].

Invitation letters were sent to the school principals of participating schools to explain the purpose of this study. After obtaining consent from schools, the hard copies of questionnaires were sent to each participating school according to the number of students in each class of the selected grade(s) between P.4 and P. 6 for primary schools and F.1 and F.3 for secondary schools. All P.4 to P.6 and F.1 to F.3 students were considered as potentially eligible participants. Schools were given the option to distribute either hard copies during class time or electronic copies of the survey, depending on their class arrangements, which were influenced by the COVID‐19 pandemic and case.

Students' participation was voluntary. Parent consent form templates were provided to schools, and secondary school students were encouraged to use the information provided in the template to notify their parents about the survey, while primary school students were required to use the form to obtain prior consent from their parents or guardians for their children participating in the survey. All participants were clearly informed of the study's confidential nature, and the school administration had no authority to access the questionnaires. Moreover, to ensure the accuracy of the data, we constructed the questionnaire using plain language supplemented with examples and visual materials to facilitate understanding. Teachers were responsible for providing standardized guidance to address participants' inquiries. A total of 2150 students were invited to participate, of which 1541 completed the survey, resulting in a response rate of 71.7% (Supporting Information Figure 1).

### 2.3 Survey Instrument

A self‐administered questionnaire on health behaviors was employed for data collection. For the secondary school students, the questionnaire was divided into three main parts with a total of 17 questions: demographic information (sex, age, and socioeconomic status), self‐perceived current weight, and lifestyle habits and health behaviors (unhealthy dietary, sedentary time, physical activity level etc.). The questionnaire for primary school students was a replica of the questionnaire for secondary school students, with the perceived overweight question deleted.

Due to the insufficient knowledge and unwillingness to disclose relevant information of children and adolescents, gathering precise information about their socioeconomic status (SES) from them might be difficult. Therefore, the Family Affluence Scale was adopted as a proxy to measure the SES status of children and adolescents [8, 9]. Questions such as ownership of private bedroom, ownership of cars (household), number of household computers, and travel history in the past 12 months were included. A composite score was calculated based on the answers of these 4 items: Low SES (score = 0–3), Medium SES(score = 4, 5), and High SES (score = 6, 7) indicated low, medium, and high affluence respectively [9], as recommended and keep consistent with previous studies [9, 10, 11].

The measurement of key covariates regarding lifestyle habits and health behaviors was adapted from the Global School‐based Student Health Survey (GSHS) developed by the WHO, including domains in physical activity and screen exposure [12]. This questionnaire was widely adopted in previous studies among the Chinese population and demonstrated a good validity and applicability [13, 14, 15]. Participants were required to report their physical activities and screen time spent watching television (TV)/playing video games/using social media over the past 7 days. Physical activity was defined as an average of at least 60 min of moderate to vigorous activity per day based on recommendations from the WHO [16]. Furthermore, participants who reported an average of two or more hours of daily screen exposure to TV, video games, or social media during weekdays were categorized as having excessive screen time exposure, as per established guidelines [17].

### 2.4 Outcome Variables

The measurement tools for the outcomes of interest in this study, including fruit and vegetable intake, unhealthy food consumption, and skipping breakfast, were adapted from the GSHS [12]. Participants were required to report their daily fruit and vegetable intake for the previous 7 days. Those who fail to meet any of the two recommendations from the Center for Health Protection in Hong Kong were considered as inadequate fruit or vegetable intake: (1) primary students are recommended to eat at least 1 bowl (2 servings) of vegetables, while secondary students are recommended to eat at least 1.5 bowls (3 servings) daily; (2) At least one bowl (2 servings) of fruits for both students [18, 19].

The habit of breakfast consumption was measured by an item that required respondents to answer how many days they had their breakfast from six options: (1) not sure, (2) 0 days, (3) 1 to 2 days, (4) 3 to 4 days, (5) 5‐6 days, and (6) 7 days of breakfast. For analytical purposes, participants who reported skipping breakfast at least once were classified as having a habit of skipping breakfast.

As for the consumption of unhealthy food, respondents were required to answer the consumption frequency of the following seven types of unhealthy food items: (1) crisps or other snacks, (2) chocolate or candies, (3) desserts, ice cream, cake, tart, (4) soft drinks, (5) carton‐packed juice, lemon tea or other sugary drinks, (6) fried food, and (7) processed or preserved meat. Each item provides 4 options: (1) No, (2) 1–3 times in 7 days, (3) 4–6 times in 7 days, and (4) Once or more a day. Participants who consumed any of the above food items at least 4 times per week were considered to have unhealthy food consumption, defined using the 3rd quartile frequency from a survey on snack consumption among children in Hong Kong [20].

### 2.5 Statistical Analysis

To conclude the participants' characteristics and the variable scores, descriptive analyzes were conducted by analyzing entered data using the IBM Statistical Package for Social Sciences (SPSS) software version 26.0. The pairwise deletion approach was employed to handle the missing data, and only the specific cases with missing values for all the outcomes of interest were excluded from the analysis [21]. The association between breakfast skipping, unhealthy food consumption, unhealthy diet, perceived weight gain, SES, gender, time spent on TV, games, and social media were evaluated using multivariate logistic regression with a threshold of two‐sided *p* < 0.05 to determine statistical significance. The variance inflation factor (VIF) test was used to examine the multicollinearity between variables, and variables with a VIF greater than 5 were excluded from the multivariate model [22]. A multivariate logistic regression model utilizing multiple imputation with chained equations for handling missing values was employed as a sensitivity analysis to validate the robustness of the findings.

## 3 Results

### 3.1 Participant Characteristics

A total of 1541 participants were included in this study, with 762 responses from primary school students and 779 responses from secondary school students (Table 1). Among all the respondents, females were the majority in both primary (53.3%, *N* = 403) and secondary (51.6%, *N* = 401) school students. Students with the low SES were the dominant group in both primary (74.1%, *N* = 553) and secondary (74.9%, *N* = 573) school students. A large proportion of primary school and secondary school students did not have adequate intake of fruits or vegetables (primary: 81.5%, *N* = 583, secondary 89.5%, *N* = 662). Most of them reported not consuming unhealthy food (primary school: 53.6%, *N* = 378; secondary: 50.8%, *N* = 375) and did not skip breakfast (primary school: 81.4%, *N* = 575; secondary: 57.2%, *N* = 403). Details of the socio‐demographic information are presented in Table 1. A summary of the missing values for each variable was presented in the Supporting Information Table 1.

**Table 1:** Participant characteristics among primary and secondary school students.

	Total Participants (*N* = 1541)	Primary school students (*N* = 762)	Secondary school students (*N* = 779)
**Sex** *			
Female	52.4% (804)	53.3% (403)	51.6% (401)
Male	47.6% (729)	46.7% (353)	48.4% (376)
**Socioeconomic status** *			
Low	74.5% (1126)	74.1% (553)	74.9% (573)
Medium	23.4% (353)	23.6% (176)	23.1% (177)
High	2.1% (32)	2.3% (17)	2.0% (15)
**Inadequate intake of fruits or vegetables** *			
No	14.4% (210)	18.5% (132)	10.5% (78)
Yes	85.6% (1245)	81.5% (583)	89.5% (662)
**Unhealthy food consumption** *			
No	52.2% (753)	53.6% (378)	50.8% (375)
Yes	47.8% (690)	46.4% (327)	49.2% (363)
**Breakfast skipping** *			
No	69.3% (978)	81.4% (575)	57.2% (403)
Yes	30.7% (433)	18.6% (131)	42.8% (302)
**Self‐perceived current weight** *			
Normal	42.1% (312)		42.1% (312)
Underweight	20.4% (151)		20.4% (151)
Overweight	37.5% (278)		37.5% (278)
**Any physical activity** *			
No	17.0% (252)	14.0% (102)	20.0% (150)
Yes	83.0% (1230)	86.0% (629)	80.0% (601)
**Time spent on TV** *			
Less than 2 h	47.0% (685)	60.7% (434)	33.8% (251)
2 h or more	53.0% (773)	39.3% (281)	66.2% (492)
**Time spent on games** *			
Less than 2 h	57.9% (843)	70.1% (503)	46.1% (340)
2 h or more	42.1% (613)	29.9% (215)	53.9% (398)
**Time spent on social media** *			
Less than 2 h	69.6% (1019)	83.2% (600)	56.4% (419)
2 h or more	30.4% (445)	16.8% (121)	43.6% (324)

* Missing values were excluded in prevalence calculations; Inadequate intake of fruits or vegetables: less than 2 servings of vegetables or 2 servings of fruits daily; Unhealthy food consumption: consumed any of the following items at least 4 times per week: (1) crisps or other snacks, (2) chocolate or candies, (3) desserts, ice cream, cake, tart, (4) soft drinks, (5) carton‐packed juice, lemon tea or other sugary drinks, (6) fried food, and (7) processed or preserved meat; Breakfast Skipping: Skipping breakfast at least once in the previous 7 days; Physical activity: an average of at least 60 min of moderate to vigorous activity per day; TV: Television.

**Figure 1:**
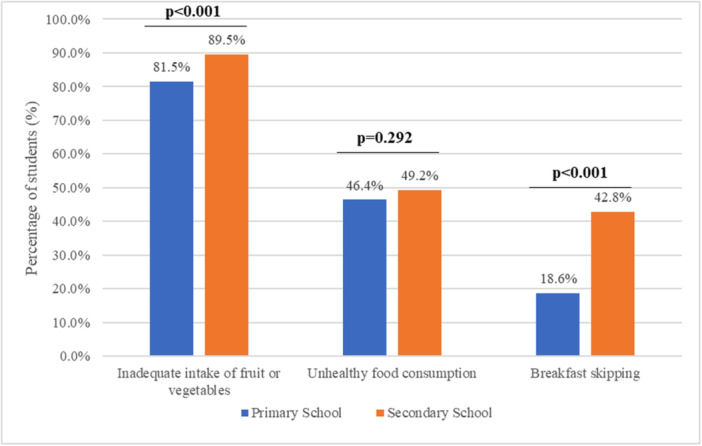
Comparison of the prevalence of unhealthy dietary habits between primary and secondary school students.

Further analysis revealed a difference in the prevalence of unhealthy dietary habits between primary and secondary school students. A significantly higher prevalence of inadequate fruit or vegetable intake (*p* < 0.001), as well as breakfast skipping behavior (*p* < 0.001), was observed among secondary school students compared to primary school students (Table 1; Figure 1).

### 3.2 Prevalence of Unhealthy Dietary Habits

**Figure 2:**
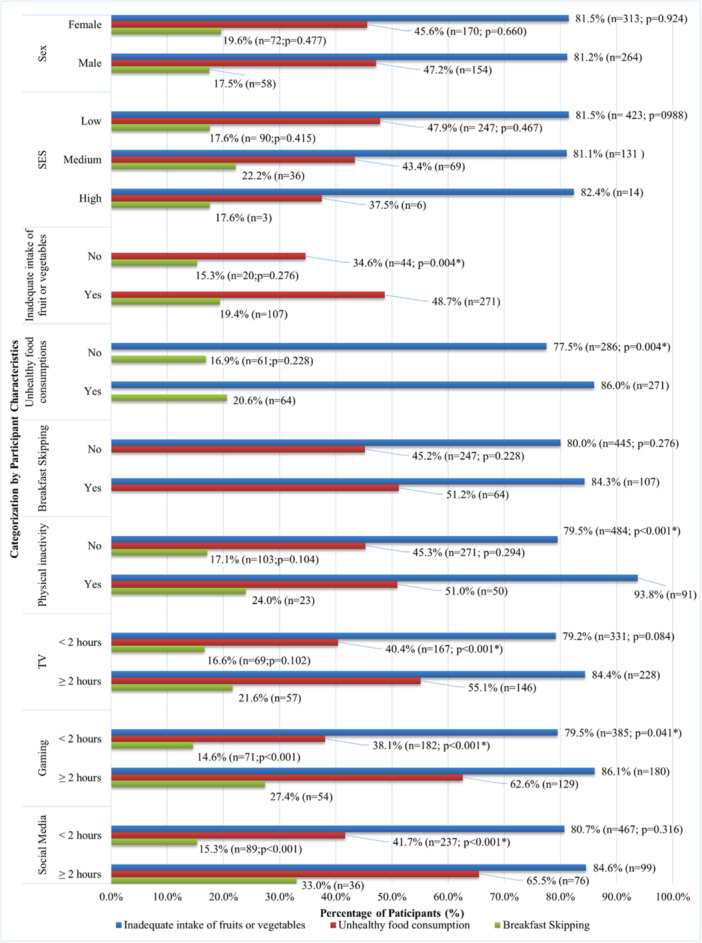
Prevalence of unhealthy dietary habits among primary school students by subgroup. *p < 0.05; Inadequate intake of fruits or vegetables: less than 2 servings of vegetables or 2 servings of fruits daily; Unhealthy food consumption: consumed any of the following items at least four times per week: (1) crisps or other snacks, (2) chocolate or candies, (3) desserts, ice cream, cake, tart, (4) soft drinks, (5) carton‐packed juice, lemon tea or other sugary drinks, (6) fried food, and (7) processed or preserved meat; Breakfast Skipping: Skipping breakfast at least once in the previous 7 days; Physical activity: an average of at least 60 min of moderate to vigorous activity per day.

In terms of primary school students (Figure 2), students who had unhealthy food consumption (86.0% vs. 77.5%, *p* = 0.004), were physically inactive (93.8% vs. 79.5%, *p* < 0.001), and spent more than 2 h on gaming (86.1% vs. 79.5%, *p* = 0.041) was found to have a higher prevalence of inadequate intake of fruits or vegetables. Conversely, a higher prevalence of unhealthy food consumption was found in students with inadequate intake of fruits or vegetables (48.8% vs. 34.6%, *p* = 0.004), also in students who spent more than 2 h on TV (55.1% vs. 40.4%, *p* < 0.001), gaming (62.6% vs. 38.1%, *p* < 0.001), and social media (65.5% vs. 41.7%, *p* < 0.001). In addition, a higher prevalence of breakfast skipping was reported among students who spent more than 2 h on gaming (27.4% vs.14.6%, *p* < 0.001) and social media (33.0% vs. 15.3%, *p* < 0.001).

**Figure 3:**
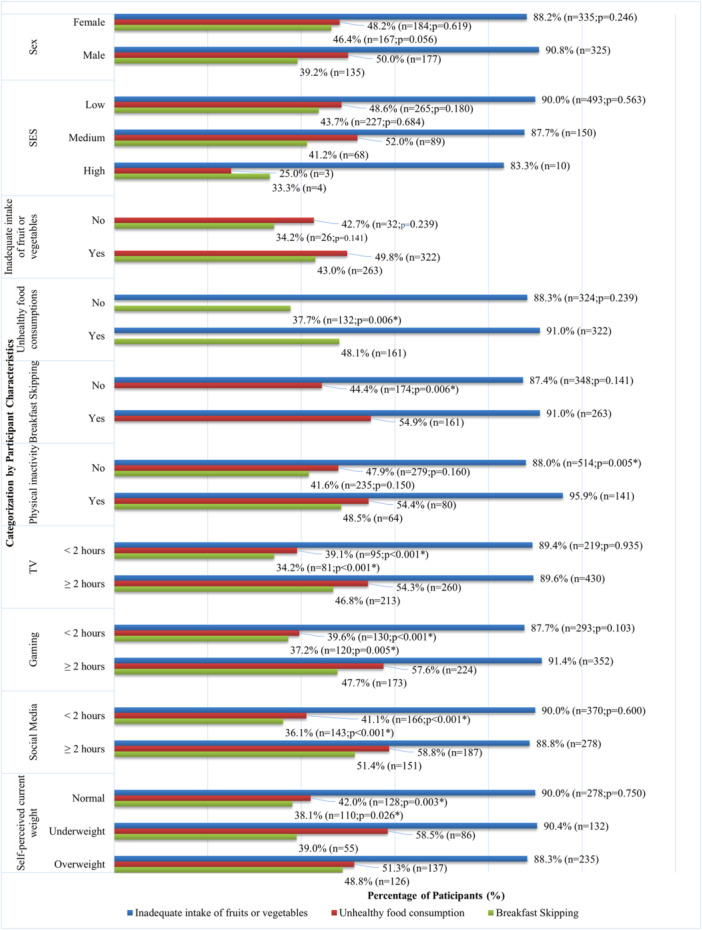
Prevalence of unhealthy dietary habits among secondary school students by subgroup. *p < 0.05; Inadequate intake of fruits or vegetables: less than two servings of vegetables or two servings of fruits daily; Unhealthy food consumption: consumed any of the following items at least four times per week: (1) crisps or other snacks, (2) chocolate or candies, (3) desserts, ice cream, cake, tart, (4) soft drinks, (5) carton‐packed juice, lemon tea or other sugary drinks, (6) fried food, and (7) processed or preserved meat; Breakfast Skipping: Skipping breakfast at least once in the previous 7 days; Physical activity: an average of at least 60 min of moderate to vigorous activity per day.

In terms of secondary school students (Figure 3), a higher prevalence of inadequate intake of fruits or vegetables was only observed in students who were physically inactive (95.9% vs. 88.0%, *p* = 0.005). Regarding unhealthy food consumption, a higher prevalence was found in students who skipped breakfast (54.9% vs. 44.4%, *p* = 0.006), self‐perceived as underweight (58.5%), or overweight (51.3% vs 42.0% for normal weight, *p* = 0.003), spent more than 2 h in TV (54.3% vs. 39.1%, *p* < 0.001), gaming (57.6% vs. 39.6%, *p* < 0.001), and social media (58.8% vs. 41.1%, *p* < 0.001). Similarly, a higher prevalence of breakfast skipping was reported in students with unhealthy food consumption (48.1% vs. 37.7%, *p* = 0.006), and students who perceived themselves as underweight (39.0%) or overweight (48.8% vs. 38.1% for normal weight, *p* = 0.026), spent more than 2 h in TV (46.8% vs. 34.2%, *p* = 0.001), gaming (47.7% vs. 37.2%, *p* = 0.005), and social media (51.4% vs. 36.1%, *p* < 0.001).

### 3.3 Associated Risk Factors

The VIF tests for each multivariate logistic regression model are presented in Supporting Information Table 2, and no multicollinearity was observed (VIF > 5) among the variables. Summary of factors significantly associated with unhealthy dietary habits for both primary and secondary school students are summarized in Supporting Information Figures 2–3.

Among the primary school students, when the inadequate intake of fruits or vegetables was the dependent variable, participants who engaged in physical inactivity (aOR: 3.26, 95% CI: 1.37–7.76, *p* = 0.008) and unhealthy food consumption (aOR = 1.67, 95%CI: 1.08–2.58, *p* = 0.020) were more likely to have inadequate intake of fruits or vegetables (Table 2). However, inadequate intake of fruits or vegetables was the associated risk factor of unhealthy food consumption (aOR: 1.67, 95% CI: 1.08–2.58, *p* = 0.020). Moreover, 2 h or more spent on games (aOR: 2.24, 95% CI: 1.46–3.44, *p* < 0.001) and social media (aOR: 2.01, 95% CI: 1.20–3.38, *p* = 0.008) were also associated with unhealthy food consumption. Similarly, breakfast skipping was also associated with spending 2 h or more on games (aOR: 1.94, 95% CI: 1.16–3.27, *p* = 0.012) and social media (aOR: 2.07, 95% CI: 1.19–3.63, *p* = 0.011).

**Table 2:** Factors associated with unhealthy dietary habits among primary school students.

	Inadequate intake of fruits or vegetables			Unhealthy food consumption			Breakfast skipping		
	aOR	95%CI	*p*‐value	aOR	95%CI	*p*‐value	aOR	95%CI	*p*‐value
**Sex**									
Female	Ref			Ref			Ref		
Male	1.01	0.67–1.52	0.954	1.01	0.72–1.41	0.966	0.97	0.64–1.48	0.892
**Socioeconomic status**									
Low	Ref			Ref			Ref		
Medium	1.03	0.63–1.67	0.913	0.85	0.57–1.27	0.430	1.29	0.79–2.10	0.310
High	1.70	0.37–7.89	0.498	0.75	0.24–2.32	0.615	0.33	0.04–2.65	0.298
**Inadequate intake of fruits or vegetables**									
No				Ref			Ref		
Yes				**1.67**	**1.08–2.58**	**0.020** *	1.22	0.69–2.16	0.489
**Unhealthy food consumption**									
No	Ref						Ref		
Yes	**1.67**	**1.08–2.58**	**0.020** *				0.97	0.62–1.50	0.885
**Breakfast Skipping**									
No	Ref			Ref					
Yes	1.22	0.69–2.15	0.498	0.96	0.62–1.49	0.838			
**Any physical activity**									
Yes	Ref			Ref			Ref		
No	**3.26**	**1.37–7.76**	**0.008** *	1.13	0.69–1.88	0.625	1.71	0.95–3.05	0.071
**Time spent on TV**									
Less than 2 h	Ref			Ref			Ref		
2 h or more	0.90	00.46–1.74	0.747	1.12	0.758–1.66	0.566	0.92	0.56–1.51	0.744
**Time spent on games**									
Less than 2 h	Ref			Ref			Ref		
2 h or more	1.20	0.68–2.12	0.536	**2.24**	**1.46–3.44**	**< 0.001** *	**1.94**	**1.16–3.27**	**0.012** *
**Time spent on social media**									
Less than 2 h	Ref			Ref			Ref		
2 h or more	1.35	0.82–2.20	0.235	**2.01**	**1.20–3.38**	**0.008** *	**2.07**	**1.19–3.63**	**0.011** *

* *p* < 0.05; inadequate intake of fruits or vegetables: less than two servings of vegetables or two servings of fruits daily; Unhealthy food consumption: consumed any of the following items at least four times per week: (1) crisps or other snacks, (2) chocolate or candies, (3) desserts, ice cream, cake, tart, (4) soft drinks, (5) carton‐packed juice, lemon tea or other sugary drinks, (6) fried food, and (7) processed or preserved meat; breakfast skipping: skipping breakfast at least once in the previous 7 days; Physical activity: an average of at least 60 min of moderate to vigorous activity per day; TV: Television; Ref: Reference group.

Similar with primary school students, among secondary school students, physical inactivity (aOR: 3.39, 95% CI: 1.31–8.74, *p* = 0.012; Table 3) was also the only risk factors associated with inadequate intake of fruits or vegetables. Meanwhile, when the unhealthy food consumption was the dependent variable, it was associated with breakfast skipping (aOR: 1.48, 95% CI: 1.05–2.07, *p* = 0.024), self‐perceived current underweight (aOR: 1.81, 95% CI: 1.15–2.83, *p* = 0.010, reference: self‐perceived current normal weight), spending 2 h or more on games (aOR: 1.56, 95% CI: 1.09–2.24, *p* = 0.015), and social media (aOR: 1.90, 95% CI: 1.31–2.74, *p* = 0.001). Setting breakfast skipping as the dependent variable, unhealthy food was also an associated risk factor (aOR: 1.48, 95% CI: 1.06–2.08, *p* = 0.023), while the other associated risk factors were self‐perceived current overweight (aOR: 1.51, 95% CI: 1.04–2.19, *p* = 0.029, reference: self‐perceived current normal weight) and spending 2 h or more on social media (aOR: 1.47, 95% CI: 1.02–2.11, *p* = 0.040).

**Table 3:** Factors associated with unhealthy dietary habits among secondary school students.

	Inadequate intake of fruits or vegetables			Unhealthy food consumption			Breakfast skipping		
	aOR	95%CI	*p*‐value	aOR	95%CI	*p*‐value	aOR	95%CI	*p*‐value
Sex									
Female	Ref			Ref			Ref		
Male	1.48	0.85–2.58	0.167	1.27	0.89–1.81	0.183	0.78	0.55–1.11	0.168
Socioeconomic status									
Low	Ref			Ref			Ref		
Medium	0.76	0.42–1.39	0.376	1.23	0.83–1.82	0.296	0.91	0.62–1.35	0.650
High	0.42	0.08–2.21	0.305	0.32	0.06–1.58	0.160	0.75	0.18–3.11	0.690
Inadequate intake of fruits or vegetables									
No				Ref			Ref		
Yes				1.22	0.70–2.13	0.478	1.50	0.86–2.62	0.158
Unhealthy food consumption									
No	Ref						Ref		
Yes	1.21	0.70–2.09	0.503				**1.48**	**1.06–2.08**	**0.023** *
Breakfast skipping									
No	Ref			Ref					
Yes	1.51	0.86–2.64	0.151	**1.48**	**1.05–2.07**	**0.024** *			
Self‐perceived current weights									
Normal	Ref			Ref			Ref		
Underweight	0.91	0.43–1.91	0.807	**1.81**	**1.15–2.83**	**0.010** *	1.00	0.64–1.57	0.997
Overweight	0.69	0.38–1.25	0.226	1.16	0.81–1.69	0.426	**1.51**	**1.04–2.19**	**0.029**
Any physical activity									
Yes	Ref			Ref			Ref		
No	**3.39**	**1.31–8.74**	**0.012** *	1.20	0.785–1.84	0.398	1.11	0.73–1.69	0.617
Time spent on TV									
Less than 2 h	Ref			Ref			Ref		
2 h or more	0.91	0.48–1.71	0.767	1.23	0.83–1.82	0.302	1.31	0.88–1.94	0.184
Time spent on games									
Less than 2 h	Ref			Ref			Ref		
2 h or more	1.39	0.78–2.49	0.269	**1.56**	**1.09–2.24**	**0.015** *	1.07	0.75–1.54	0.704
Time spent on social media									
Less than 2 h	Ref			Ref			Ref		
2 h or more	0.76	0.42–1.37	0.356	**1.90**	**1.31–2.74**	**0.001** *	**1.47**	**1.02–2.11**	**0.040** *

* *p* < 0.05; Inadequate intake of fruits or vegetables: less than three servings of vegetables or two servings of fruits daily; Unhealthy food consumption: consumed any of the following items at least four times per week: (1) crisps or other snacks, (2) chocolate or candies, (3) desserts, ice cream, cake, tart, (4) soft drinks, (5) carton‐packed juice, lemon tea or other sugary drinks, (6) fried food, and (7) processed or preserved meat; Breakfast Skipping: Skipping breakfast at least once in the previous 7 days; Physical activity: an average of at least 60 min of moderate to vigorous activity per day; TV: Television; Ref: Reference group.

### 3.4 Sensitivity Analysis

The results of the sensitivity analysis are presented in Supporting Information Tables 3 and 4, which demonstrate findings similar to the main results, further supporting the robustness of the main findings.

## 4 Discussion

### 4.1 Summary of Major Findings

Our study aims to investigate the prevalence and factors associated with unhealthy dietary habits among primary and secondary school students. There are several major findings. (1) inadequate consumption of fruit and vegetable was common among both primary and secondary school students and that it was associated with physical inactivity; (2) 18.2% of primary and 40.8% of secondary students reported breakfast skipping, and such habit was associated with longer time spent on social media and games for both age cohorts; (3) Approximately half of the respondents consumed unhealthy food, primary and secondary school students spending longer time spent on social media and video games were more likely to consume unhealthy food. Meanwhile, the increased risk was also found among secondary school students who believed they were underweight.

### 4.2 Unhealthy Dietary

Applying the Health Belief Model enables us to gain a better understanding of unhealthy dietary habits and the factors related to attitude and behavior, particularly from the perspective of perceived barriers and action cues changed during the COVID‐19 pandemic, as identified.

Firstly, this study highlighted a high prevalence of unhealthy dietary habits among the school children in Hong Kong during the COVID‐19 pandemic, providing similar findings to previous studies [23, 24], and underscoring the increased barriers for the development of healthy dietary behavior from the external environment. Study indicated the role of SES during the pandemic, children from isolated families (all members available to perform activities, like academic, work‐related, or leisure activities from home) in Brazil reportedly had healthier dietary habits than those from non‐isolated families, consuming more vegetables, raw salad, beans, and fresh fruit products. The difference in dietary habits may be attributed to higher purchasing power among isolated families and distribution challenges that made fresh and natural foods less accessible to lower‐class families [23, 25]. Likewise, a study from Italy further validated this hypothesis, emphasizing that food consumption patterns are influenced by external pressures and that SES disparities played a crucial role in shaping unhealthy dietary patterns during the pandemic [26], which is a phenomenon not confined solely to the school‐age population.

Furthermore, the results indicated that students engaged in sedentary activities, including physical inactivity and screen time, exhibited a higher propensity for unhealthy dietary habits. A study from the U.S. identified that high school students who watched TV more than 2 h per day were more likely to be overweight, be sedentary, and consume fewer fruits and vegetables [27]. The underlying reasons for this association may be attributed to the increased use of online entertainment during the pandemic. Considering Hong Kong's limited living space [28], the internet is often regarded as an auxiliary form of daily entertainment for schoolchildren during the pandemic [29], replacing indoor and outdoor activities, which increases the exposure to industrialized food marketing among schoolchildren [30]. Moreover, emotional eating during the pandemic may explain the prevalence of unhealthy dietary habits observed. An earlier survey conducted in Hong Kong indicated that 62% of children consumed more food, including high‐energy, high‐sugar items, due to boredom [31]. Similarly, research conducted in various regions has highlighted that feelings of concern and anxiety resulting from staying at home tend to intensify desires for comfort eating, particularly involving foods that are high in fat, sugar, or calorie‐dense foods [32, 33]. These observations highlight a significant concern and its detrimental effects. On one hand, the adverse outcomes, such as obesity, from unhealthy dietary habits have been well‐demonstrated [34, 35, 36, 37]. On the other hand, the behavioral patterns established during childhood may persist into adulthood, underscoring the necessity for interventions to eliminate the detrimental habits formed during the COVID‐19 pandemic [38].

It is noteworthy that this study did not observe gender differences in unhealthy dietary habits, representing an inconsistency with previous studies. For instance, research from Japan found that female adolescents were less likely than male adolescents to exhibit unhealthy eating habits [39]. Likewise, studies from Africa and the Middle East also noted the hypothesis of gender differences regarding unhealthy dietary habits [40, 41]. This difference may be partially attributable to stricter parental supervision and parenting during the COVID‐19 pandemic [42, 43], as parents had more opportunities and time spent with their teenagers.

### 4.3 Self‐Perceived Current Weight and Unhealthy Dietary Habits

This study indicated that over 55% of secondary school students perceived their weights as overweight or underweight, highlighting a concerning issue of weight perception among school children in Hong Kong. A previous study from Spain indicated that following the COVID‐19 pandemic, body dissatisfaction among children and adolescents has increased significantly, reaching approximately three times the levels observed before the pandemic [44]. In addition, only 26% and 21% of 6–13 years old boys and girls have positive self‐perceived physical fitness (SPPF) during the period of COVID‐19 restrictions [45]. The reason behind the trend may be linked to the social media usage during the pandemic. Evidence indicates that high social media usage is a key factor contributing to body dissatisfaction among children and adolescents [46], and this internet activity reliance intensified during the pandemic [29]. This phenomenon is concerning, as perception of weight abnormality, which may lead to perceived weight stigma and higher levels of psychological distress [47, 48], compared with normal‐weight students. Our study found an association between self‐perceived overweight and increased risk of skipping breakfast, it is possible that the adolescents attempted to lose weight by skipping breakfast as school students may have a misconception that skipping breakfast could decrease their weight, BMI, or help build body shape [49]. However, breakfast is the most crucial meal of the day for children: Breakfast in the morning was positively associated with memory recall, children's performance at school, mood, and cognitive function, as well as a decreased risk in obesity and a positive impact on BMI [50]. In contrast, skipping breakfast on an irregular basis was linked to a wide range of effects including depression, decreased happiness, PTSD, loneliness, short sleep, extended sleep, sleep problem, restless sleep, as well as poor academic performance [51].

Furthermore, we found that underweight students were more likely to eat unhealthy food in daily. A Chilean study identified that underweight male people were more likely to consume unhealthy food [52]. They may believe that unhealthy food consumption was a good way to increase their weight or BMI. Similar findings were also found in India that underweight students were more prone to unhealthy behaviors, such as having unhealthy dietary [53].

### 4.4 Limitation

Although this study provided valuable insights into the unhealthy dietary behaviors of primary and secondary school students in Hong Kong during the COVID‐19 pandemic by utilizing a large sample size, several limitations should be acknowledged. Firstly, the data for this study are based on self‐reported information, which may introduce potential biases, particularly social desirability bias and recall bias. Likewise, the potential of measurement error may be introduced by the self‐administered reporting, especially among younger children. However, to minimize the occurrence of such biases, additional efforts were undertaken, including emphasizing the confidentiality of the study and providing visual materials to support the accuracy of data reporting. Secondly, although using household or parental SES to represent children's SES has been a common approach, the actual effects may be obscured by using proxy SES measures. Thirdly, this study consists of schoolchildren from Hong Kong and is grounded in an eastern cultural context; the generalizability must be cautiously evaluated when extending the findings to other regions. Fourthly, one limitation of this study is the absence of a pilot test. No preliminary testing of the questionnaire was conducted, which may slightly affect the refinement of the instrument and the overall robustness of the data collection process. Future studies would benefit from incorporating a pilot phase to gather feedback and make necessary adjustments to enhance the quality of the research. Fifthly, it is worth noting the potential self‐selection bias, as students and schools with higher health awareness may be more likely to participate, which may lead to an underestimation of unhealthy dietary habits. Lastly, a cross‐sectional design was employed in this study, which precludes causal inferences; further study should utilize longitudinal data to validate potential causal inference among different covariates identified.

### 4.5 Implications

The high prevalence of unhealthy dietary habits among school children in Hong Kong is concerning. Our findings suggest that physical inactivity is associated with a higher risk of these habits, which may increase the risk of obesity in primary and secondary students. Given that multiple unhealthy habits were found to be interrelated, a comprehensive approach to promoting healthy habits through education is needed. Weight management is particularly important for students' overall health. Overweight students were less likely to eat breakfast daily in an effort to lose weight, while underweight students were more likely to consume unhealthy foods in an attempt to gain weight. Therefore, a health education program on weight management and weight perception awareness is crucial for children and adolescents. Additionally, given the high prevalence of inadequate fruit and vegetable intake, unhealthy food consumption, and skipping breakfast, particularly among secondary school students, existing programs such as EatSmart@school.hk could consider enhancing and regulating the food patterns and combinations provided by lunch vendors to better address this issue [54]. Furthermore, policymakers may explore implementing a School Breakfast Program to promote healthy lifestyles [55, 56, 57]. Such interventions can help students achieve a healthy life and avoid unhealthy habits. Future research could investigate the effectiveness of various nutrition education interventions in promoting healthy eating habits and preventing unhealthy ones among children in the post‐COVID‐19 pandemic period of Hong Kong.

## Author Contributions

Junjie Huang conceptualized and supervised the study and drafted the initial article. Sze Chai Chan was responsible for data curation, formal analysis and drafted the initial article. Wing Sze Pang, Fung Yu Mak, and Yat Ching Fung drafted the initial article. Vera MW Keung, Calvin KM Cheung, Vincent TC Lau, and Amelia SC Lo were responsible for data acquisition, reviewed and revised the article. Claire Chenwen Zhong, Lancelot WH Mui and Albert Lee reviewed and revised the article. Martin CS Wong conceptualized and designed the study, reviewed and revised the article. All authors approved the final article as submitted and agree to be accountable for all aspects of the work.

## Ethics Statement

Ethical approval was obtained from the Survey and Behavioral Research Ethics Committee, The Chinese University of Hong Kong (No. SBRE‐21‐0052).

## Conflicts of Interest

No conflicts of interest to be declared by co‐authors for this study.

## Transparency Statement

The lead author Junjie Huang, Claire Chenwen Zhong, Martin C. S. Wong affirms that this article is an honest, accurate, and transparent account of the study being reported; that no important aspects of the study have been omitted; and that any discrepancies from the study as planned (and, if relevant, registered) have been explained.

## Supporting information

Go_Smart_diet_R2_Supplementary_Material_12Nov2025.

## Data Availability

The data that supports findings of this study are available from the corresponding author, upon reasonable request.
