# Supplementary material for: Prevalence and Risk Factors for Unhealthy Dietary Habits Among School Children in Hong Kong During COVID‐19 Pandemic: A Cross‐Sectional Study
Source: Health Sci Rep. 2026 Jan 19;9(1):e71746. doi: 10.1002/hsr2.71746 (PMC12816883; doi:10.1002/hsr2.71746)

Supplementary Table 1. Summary of Missing Value

|  | Primary School Students (N=762) | Secondary School Student (N=779) |
| --- | --- | --- |
| **Sex** | 0.8% (6) | 0.3% (2) |
| **Socioeconomic Status** | 2.1% (16) | 1.8% (14) |
| **Inadequate Intake of Fruits or Vegetables** | 6.2% (47) | 5.0% (39) |
| **Unhealthy Food Consumption** | 7.5% (57) | 5.3% (41) |
| **Breakfast Skipping** | 7.3% (56) | 9.5% (74) |
| **Self-perceived Current Weight** | Not applicable | 4.9% (38) |
| **Any Physical activity** | 4.1% (31) | 3.6% (28) |
| **Time Spent on TV** | 6.2% (47) | 4.6% (36) |
| **Time Spent on Games** | 5.8% (44) | 5.3% (41) |
| **Time Sent on Social Media** | 5.4% (41) | 4.6% (36) |

Inadequate intake of fruits or vegetables: less than 2 servings of vegetables or 2 servings of fruits daily; Unhealthy food consumption: consumed any of the following items at least 4 times per week:(1) crisps or other snacks, (2) chocolate or candies, (3) desserts, ice cream, cake, tart, (4) soft drinks, (5) carton-packed juice, lemon tea or other sugary drinks, (6) fried food, and (7) processed or preserved meat; Breakfast Skipping: Skipping breakfast at least once in the previous seven days; Physical activity: an average of at least 60 minutes of moderate to vigorous activity per day.

Supplementary Table 2. Multicollinearity test

1. Multicollinearity test for factors associated with inadequate intake of fruits or vegetable among primary school student

|  | **GVIF** | **Df** | **GVIF^(1/(2*Df))** |
| --- | --- | --- | --- |
| Gender | 1.007913 | 1 | 1.003949 |
| Socio-economic status | 1.029657 | 2 | 1.007333 |
| Physical activity | 1.012559 | 1 | 1.00626 |
| Time spent on TV | 1.305572 | 1 | 1.142616 |
| Time spent on games | 1.413223 | 1 | 1.18879 |
| Time spent on social media | 1.291687 | 1 | 1.136524 |
| Skipping breakfast | 1.042223 | 1 | 1.020893 |
| Unhealthy food consumption | 1.065283 | 1 | 1.032126 |

1. Multicollinearity test for factors associated with unhealth food consumption among primary school student

|  | **GVIF** | **Df** | **GVIF^(1/(2*Df))** |
| --- | --- | --- | --- |
| Gender | 1.008677 | 1 | 1.004329 |
| Socio-economic status | 1.038053 | 2 | 1.00938 |
| Physical activity | 1.037759 | 1 | 1.018705 |
| Time spent on TV | 1.306496 | 1 | 1.143021 |
| Time spent on games | 1.342046 | 1 | 1.158467 |
| Time spent on social media | 1.191585 | 1 | 1.091597 |
| Skipping breakfast | 1.048107 | 1 | 1.023771 |
| Inadequate intake of fruits or vegetable | 1.017051 | 1 | 1.00849 |

1. Multicollinearity test for factors associated with skipping breakfast among primary school student

|  | **GVIF** | **Df** | **GVIF^(1/(2*Df))** |
| --- | --- | --- | --- |
| Gender | 1.010395 | 1 | 1.005184 |
| Socio-economic status | 1.034002 | 2 | 1.008394 |
| Physical activity | 1.048583 | 1 | 1.024003 |
| Time spent on TV | 1.379995 | 1 | 1.174732 |
| Time spent on games | 1.459602 | 1 | 1.20814 |
| Time spent on social media | 1.283322 | 1 | 1.132838 |
| Inadequate intake of fruits or vegetable | 1.029826 | 1 | 1.014804 |
| Unhealthy food consumption | 1.09187 | 1 | 1.044926 |

1. Multicollinearity test for factors associated with inadequate intake of fruits or vegetable among secondary school student

|  | **GVIF** | **Df** | **GVIF^(1/(2*Df))** |
| --- | --- | --- | --- |
| Gender | 1.116469 | 1 | 1.056631 |
| Socio-economic status | 1.049429 | 2 | 1.012134 |
| Physical activity | 1.020913 | 1 | 1.010403 |
| Time spent on TV | 1.294347 | 1 | 1.137694 |
| Time spent on games | 1.253048 | 1 | 1.119396 |
| Time spent on social media | 1.303948 | 1 | 1.141905 |
| Self-perceived current weight | 1.080315 | 2 | 1.019501 |
| Skipping breakfast | 1.053699 | 1 | 1.026498 |
| Unhealthy food consumption | 1.090131 | 1 | 1.044093 |

1. Multicollinearity test for factors associated with unhealth food consumption among secondary school student

|  | **GVIF** | **Df** | **GVIF^(1/(2*Df))** |
| --- | --- | --- | --- |
| Gender | 1.146404 | 1 | 1.070703 |
| Socio-economic status | 1.023322 | 2 | 1.00578 |
| Physical activity | 1.05604 | 1 | 1.027638 |
| Time spent on TV | 1.241038 | 1 | 1.114019 |
| Time spent on games | 1.192578 | 1 | 1.092052 |
| Time spent on social media | 1.223101 | 1 | 1.105939 |
| Self-perceived current weight | 1.061685 | 2 | 1.015077 |
| Skipping breakfast | 1.034655 | 1 | 1.01718 |
| Inadequate intake of fruits or vegetable | 1.02415 | 1 | 1.012003 |

1. Multicollinearity test for factors associated with skipping breakfast among primary school student

|  | **GVIF** | **Df** | **GVIF^(1/(2*Df))** |
| --- | --- | --- | --- |
| Gender | 1.137144 | 1 | 1.066369 |
| Socio-economic status | 1.034036 | 2 | 1.008403 |
| Physical activity | 1.055781 | 1 | 1.027512 |
| Time spent on TV | 1.253925 | 1 | 1.119788 |
| Time spent on games | 1.231337 | 1 | 1.109656 |
| Time spent on social media | 1.236977 | 1 | 1.112195 |
| Self-perceived current weight | 1.062567 | 2 | 1.015288 |
| Inadequate intake of fruits or vegetable | 1.024952 | 1 | 1.012399 |
| Unhealthy food consumption | 1.074364 | 1 | 1.036515 |

Supplementary Table 3. Sensitivity Analysis Result of Factors Associated with Unhealthy Dietary Habits among Primary School Students

|  | Inadequate intake of fruits or vegetables | | | Unhealthy food consumption | | | Breakfast skipping | | |  |
| --- | --- | --- | --- | --- | --- | --- | --- | --- | --- | --- |
|  | aOR | 95%CI | p- value | aOR | 95%CI | p- value | aOR | 95%CI | p- value |  |
| **Sex** |  | | |  | | |  | | |  |
| Female | Ref | | | Ref | | | Ref | | |  |
| Male | 0.91 | 0.60-1.37 | 0.656 | 1.1 | 0.80-1.52 | 0.547 | 0.85 | 0.57-1.26 | 0.425 |  |
| **Socioeconomic status** |  | | |  | | |  | | |  |
| Low | Ref | | | Ref | | | Ref | | |  |
| Medium | 1.05 | 0.66-1.68 | 0.828 | 0.77 | 0.54-1.11 | 0.160 | 1.36 | 0.82-2.25 | 0.229 |  |
| High | 1.25 | 0.34-4.53 | 0.737 | 0.64 | 0.21-1.93 | 0.429 | 0.96 | 0.26-3.54 | 0.957 |  |
| **Inadequate intake of fruits or vegetables** |  | | |  | | |  | | |  |
| No |  |  |  | Ref | | | Ref | | |  |
| Yes |  |  |  | **1.65** | **1.10-2.47** | **0.015*** | 1.20 | 0.69-2.06 | 0.519 |  |
| **Unhealthy food consumption** |  | | |  | | |  | | |  |
| No | Ref | | |  |  |  | Ref | | |  |
| Yes | **1.65** | **1.10-2.48** | **0.015*** |  |  |  | 0.99 | 0.63-1.56 | 0.964 |  |
| **Breakfast Skipping** |  | | |  | | |  | | |  |
| No | Ref | | | Ref | | |  |  |  |  |
| Yes | 1.18 | 0.69-2.04 | 0.539 | 0.98 | 0.62-1.55 | 0.941 |  |  |  |  |
| **Any physical activity** |  | | |  | | |  | | |  |
| Yes | Ref | | | Ref | | | Ref | | |  |
| No | **4.03** | **1.66-9.79** | **0.002*** | 1.22 | 0.78-1.91 | 0.375 | **1.72** | **1.03-2.90** | **0.040*** |  |
| **Time spent on TV** |  | | |  | | |  | | |  |
| Less than 2 hours | Ref | | | Ref | | | Ref | | |  |
| 2 hours or more | 1.24 | 0.77-1.99 | 0.372 | 1.26 | 0.88-1.79 | 0.207 | 0.98 | 0.62-1.53 | 0.916 |  |
|  |  |  |  |  |  |  |  |  |  |  |
| **Time spent on games** |  |  |  |  |  |  |  |  |  |  |
| Less than 2 hours | Ref | | | Ref | | | Ref | | |  |
| 2 hours or more | 1.26 | 0.72-2.19 | 0.420 | **1.99** | **1.34-2.94** | **<0.001*** | **1.68** | **1.03-2.75** | **0.038*** |  |
|  |  |  |  |  |  |  |  |  |  |  |
| **Time spent on social media** | |  |  |  |  |  |  |  |  |  |
| Less than 2 hours | | Ref | | | Ref | | | Ref | | |
| 2 hours or more | | 0.96 | 0.52-1.77 | 0.897 | **1.78** | **1.10-2.87** | **0.018*** | **2.07** | **1.25-3.41** | **0.004*** |
|  | |  |  |  |  |  |  |  |  |  |

*p<0.05; Inadequate intake of fruits or vegetables: less than 3 servings of vegetables or 2 servings of fruits daily; Unhealthy food consumption: consumed any of the following items at least 4 times per week:(1) crisps or other snacks, (2) chocolate or candies, (3) desserts, ice cream, cake, tart, (4) soft drinks, (5) carton-packed juice, lemon tea or other sugary drinks, (6) fried food, and (7) processed or preserved meat; Breakfast Skipping: Skipping breakfast at least once in the previous seven days; Physical activity: an average of at least 60 minutes of moderate to vigorous activity per day; TV: Television; Ref: Reference group.

Supplementary Table 4. Sensitivity Analysis Result of Factors Associated with Unhealthy Dietary Habits among Secondary School Students

|  | Inadequate intake of fruits or vegetables | | | Unhealthy food consumption | | | Breakfast skipping | | |  |
| --- | --- | --- | --- | --- | --- | --- | --- | --- | --- | --- |
|  | aOR | 95%CI | p- value | aOR | 95%CI | p- value | aOR | 95%CI | p- value |  |
| Sex |  | | |  | | |  | | |  |
| Female | Ref | | | Ref | | | Ref | | |  |
| Male | 1.37 | 0.82-2.28 | 0.223 | 1.14 | 0.83-1.58 | 0.419 | 0.79 | 0.57-1.10 | 0.166 |  |
| Socioeconomic status |  | | |  | | |  | | |  |
| Low | Ref | | | Ref | | | Ref | | |  |
| Medium | 0.81 | 0.47-1.40 | 0.446 | 1.13 | 0.79-1.63 | 0.498 | 0.91 | 0.64-1.31 | 0.619 |  |
| High | 0.53 | 0.09-3.24 | 0.483 | 0.29 | 0.08-1.11. | 0.071 | 1.00 | 0.22-4.43 | > 0.999 |  |
| Inadequate intake of fruits or vegetables |  | | |  | | |  | | |  |
| No |  |  |  | Ref | | | Ref | | |  |
| Yes |  |  |  | 1.16 | 0.70-1.94 | 0.560 | 1.39 | 0.83-2.32 | 0.209 |  |
| Unhealthy food consumption |  | | |  | | |  | | |  |
| No | Ref | | |  |  |  | Ref | | |  |
| Yes | 1.16 | 0.70-1.93 | 0.562 |  |  |  | **1.37** | **1.00-1.87** | **0.047*** |  |
| Breakfast Skipping |  | | |  | | |  | | |  |
| No | Ref | | | Ref | | |  |  |  |  |
| Yes | 1.4 | 0.84-2.33 | 0.199 | **1.37** | **1.00-1.87** | **0.049*** |  |  |  |  |
| Self-perceived current weights | |  | | |  | | |  | | |
| Normal | | Ref | | | Ref | | | Ref | | |
| Underweight | | 0.92 | 0.45-1.88 | 0.825 | **1.91** | **1.16-3.14** | **0.012*** | 1.00 | 0.64-1.56 | 0.997 |
| Overweight | | 0.73 | 0.43-1.24 | 0.238 | 1.36 | 0.96-1.92 | 0.082 | 1.40 | 0.96-2.04 | 0.081 |
| Any physical activity |  | | |  | | |  | | |  |
| Yes | Ref | | | Ref | | | Ref | | |  |
| No | **3.12** | **1.33-7.30** | **0.009*** | 1.33 | 0.90-1.96 | 0.145 | 1.17 | 0.79-1.72 | 0.436 |  |
| Time spent on TV |  | | |  | | |  | | |  |
| Less than 2 hours | Ref | | | Ref | | | Ref | | |  |
| 2 hours or more | 0.93 | 0.53-1.62 | 0.798 | 1.23 | 0.84-1.78 | 0.281 | 1.34 | 0.93-1.94 | 0.118 |  |
|  |  |  |  |  |  |  |  |  |  |  |
| Time spent on games |  |  |  |  |  |  |  |  |  |  |
| Less than 2 hours | Ref | | | Ref | | | Ref | | |  |
| 2 hours or more | 1.6 | 0.94-2.75 | 0.086 | **1.55** | **1.10-2.17** | **0.012*** | 1.19 | 0.85-1.67 | 0.318 |  |
|  |  |  |  |  |  |  |  |  |  |  |
| Time spent on social media | |  |  |  |  |  |  |  |  |  |
| Less than 2 hours | | Ref | | | Ref | | | Ref | | |
| 2 hours or more | | 0.78 | 0.43-1.40 | 0.393 | **1.7** | **1.22-2.36** | **0.002*** | 1.45 | 1.02-2.05 | **0.041*** |
|  | |  |  |  |  |  |  |  |  |  |

*p<0.05; Inadequate intake of fruits or vegetables: less than 3 servings of vegetables or 2 servings of fruits daily; Unhealthy food consumption: consumed any of the following items at least 4 times per week:(1) crisps or other snacks, (2) chocolate or candies, (3) desserts, ice cream, cake, tart, (4) soft drinks, (5) carton-packed juice, lemon tea or other sugary drinks, (6) fried food, and (7) processed or preserved meat; Breakfast Skipping: Skipping breakfast at least once in the previous seven days; Physical activity: an average of at least 60 minutes of moderate to vigorous activity per day; TV: Television; Ref: Reference group.

Supplementary Figure 1. Participant Flow Chart


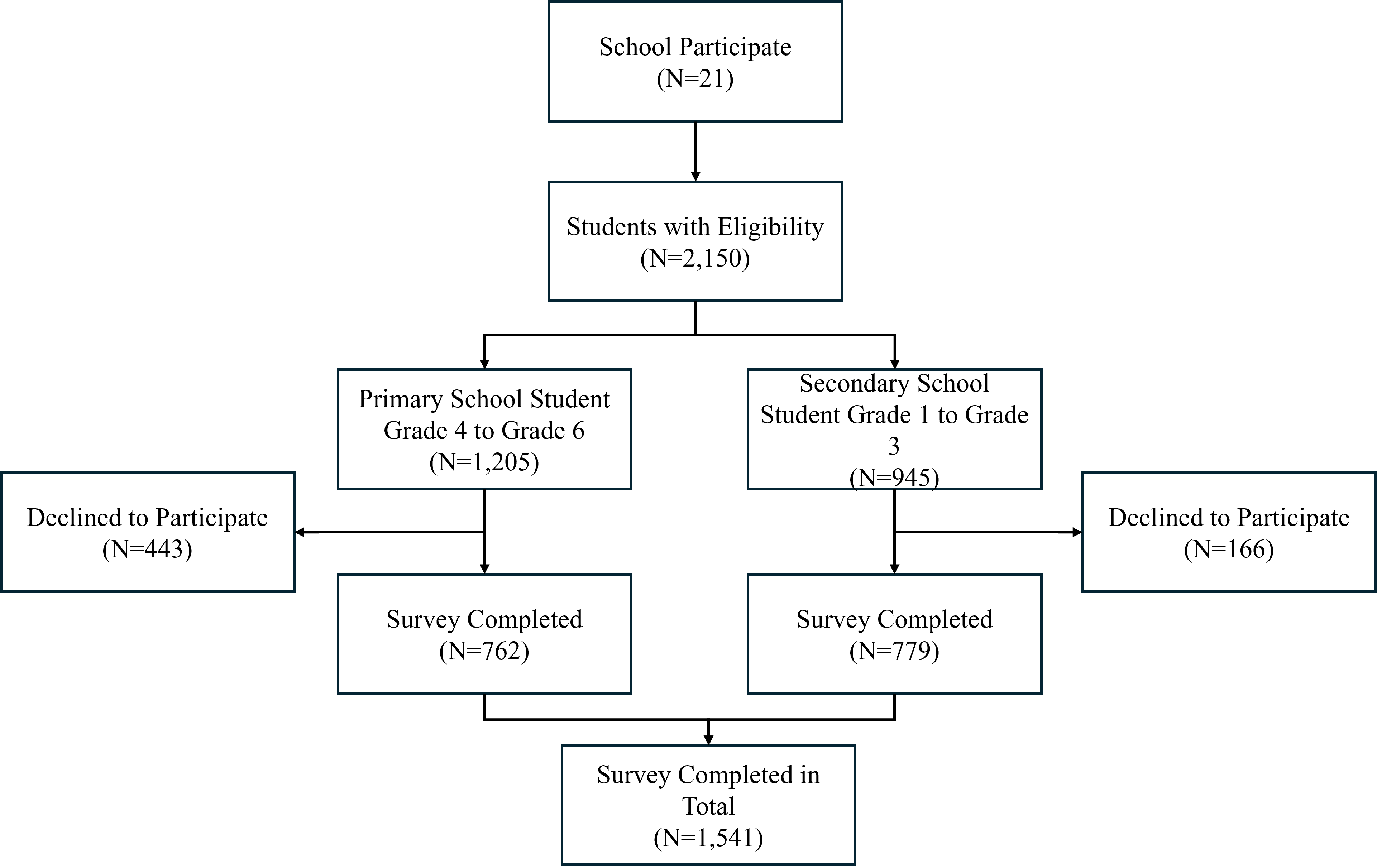


Supplementary Figure 2. Forest Plot for Summary of Key Findings among Primary School Students


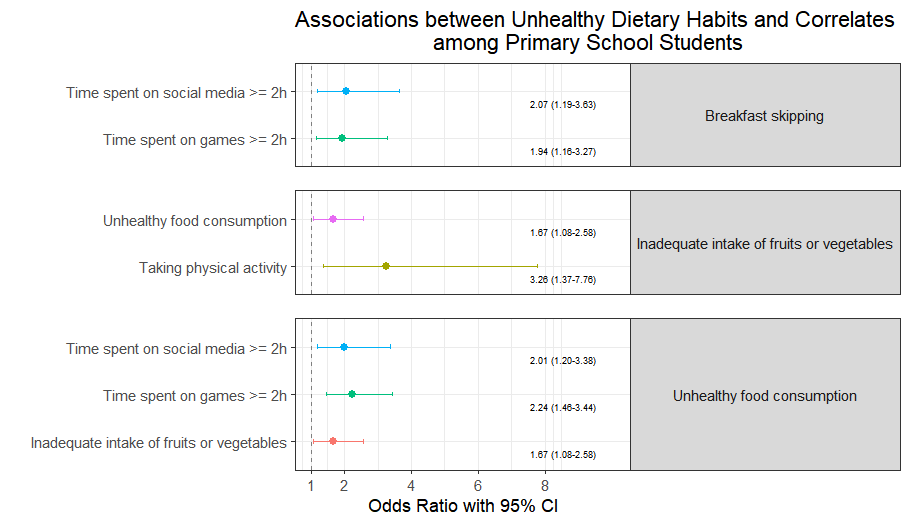


Supplementary Figure 3. Forest Plot for Summary of Key Findings among Secondary School Students


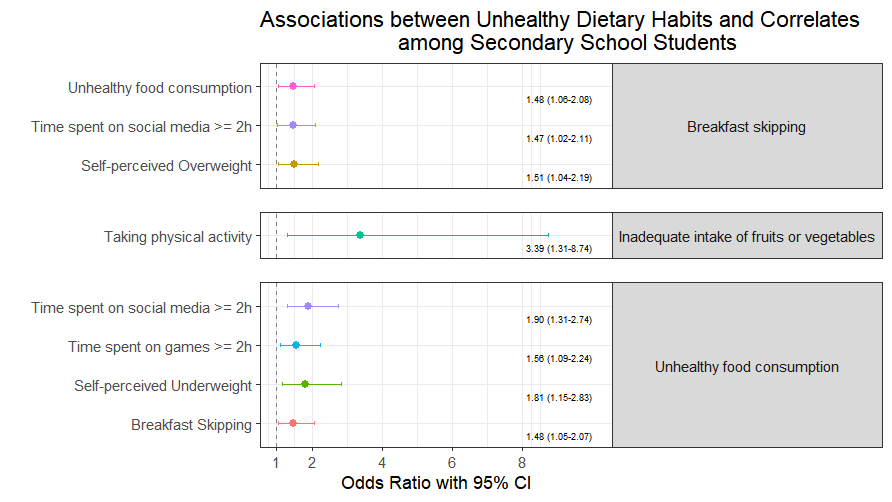

Supplement: Supplementary file 1 — Go_Smart_diet_R2_Supplementary_Material_12Nov2025. [file HSR2-9-e71746-s001.docx]
